# Nature-Based Welfare in Older Adulthood: An Eco-Appreciation Perspective

**DOI:** 10.1093/geronb/gbaf041

**Published:** 2025-02-25

**Authors:** Mali Nevo, Lia Levin

**Affiliations:** Bob Shapell School of Social Work, Tel Aviv University, Tel Aviv, Israel; Bob Shapell School of Social Work, Tel Aviv University, Tel Aviv, Israel; (Social Sciences Section)

**Keywords:** Environment, Lifestyle, Successful aging

## Abstract

**Objectives:**

This study examined the reciprocal relationship between older adults’ well-being and their accounts of human-nature relations (HNR). Guided by the tenets of the Eco-Appreciation Perspective, the question addressed was: What can be learned from older adults’ relations with nature about reciprocal nature-based welfare in older adulthood?

**Methods:**

In-depth interviews were conducted with 60 participants over the age of 65 in Israel, followed by a sequential deductive-inductive analysis of the data.

**Results:**

Four major themes were yielded: HNR as presence and being; HNR as connection; HNR as past, present, and future; and HNR as benevolence. Participants’ experiences revealed an interaction between gratitude and awareness toward HNR and an enhanced sense of well-being, purpose, meaning, and belonging. The analysis also exposed novel insights into how HNR in older adulthood can prompt mutual nature-based welfare and provide an ongoing source of comfort and resilience, both through current activities and by accessing childhood memories.

**Discussion:**

The findings are discussed in the context of eco-centric viewpoints on contemporary aging, and highlight the active role that older adults can play in deepening their connection with nature while calling on professionals in health, gerontology, social work, and community care to recognize and harness the mutual benefits of this bond.

As is widely evidenced, we are presently living in an aging society, in both relative and absolute terms (World Health Organization, 2024). One way to view older adulthood is through an anthropocentric perspective, that is solely focused on aging humans’ situation, attitudes, and demographics, while ignoring the role of nature in their lives or referring to it only insofar as it satisfies human needs (Kaida and Kaida, 2016). Another way is by applying an eco-centric perspective, that situates older adulthood in the context of human–nature relationships (HNR; Gagliardi and Piccinini, 2019). HNR are considered by many to be a significant source of inspiration, resilience, and healing for humanity and the planet (Berger and McLeod, 2006; Soga and Gaston, 2016). Accordingly, this perspective suggests that aging entails social as well as environmental aspects, thus supporting an eco-social approach to older adults’ welfare (see Author Note 1) that combines human and environmental considerations (Murphy, 2023).

Understanding aging and older adults’ welfare through an eco-social prism is all too often still limited to the narrow (albeit extremely important) field of global climate and nature-related threats (Rhoades et al., 2018). This creates a “blind spot” in the examination of facets of HNR that play out on the more immediate, subjective, individual level of older adulthood, and often masks some of HNR’s beneficial capacities, especially and specifically for older adults. In other words, it can be argued that the eco-social prism has not fully realized its wholistic potential for cultivating older adults’ welfare in the present socionatural environment. Pioneering studies in this area demonstrate that a connection with nature can be significant in older adults’ welfare. For example, research has shown that nature-based activities enhance older adults’ welfare, provide opportunities for physical activity, and decrease social isolation (e.g., Duedahl et al., 2022). Consistent therewith, Berger (2009) proposed a framework for incorporating nature into therapy with older adults and gives examples of how nature therapy can help older adults expand their outlook, recognize their strengths, and broaden their coping strategies. Studies also highlight the role of older adults’ interactions with nature in making meaning of potential stressful events, such as relocating to aged-care homes (e.g., Tsai et al., 2020). HNR can facilitate a sense of continuity, regeneration, and presence, even in present times of “extinction of experience” due to growing disconnect between humans and their environments (Beery et al., 2023).

Nonetheless, HNR studies are largely rooted in anthropocentric approaches to HNR and older adulthood (Barragan-Jason et al., 2023), wherein nature is a backdrop or scenery against which processes take place. The reciprocal elements of HNR have barely been addressed by research, and although mentioned in theory, are seldom supported by data (Ojeda et al., 2022). The present study aimed to fill part of this lacuna in knowledge, by applying the Eco-Appreciation Perspective (Nevo, 2019, 2024) toward gaining a better understanding of a possible joint process, by which older adults’ welfare may be supported by nature, and nature’s welfare may in turn be enhanced by older adults’ care.

The Eco-Appreciation Perspective involves three elements; The first, *Authentic Eco-Appreciation*, refers to distinct episodes of a genuine sense of connection with nature, whether experienced by an individual or a group, that encompass a deep recognition of mutual ecological value. These episodes can be characterized by vivid encounters with nature, arising from diverse sources, including direct or indirect, intense or subtle interactions with the natural environment. By identifying and reflecting on the intrinsic value of these experiences, eco-appreciation fosters a more palpable, mindful, and aware contact with nature; that draws attention to the possibility that both human and nonhuman features of the natural environment benefit from each other’s being. This makes room for a more inclusive and collective approach to HNR. Authentic Eco-Appreciation builds on concepts such as nature connectedness, which represents the experiential view of oneself as a member and kin of the wider natural community, while recognizing the interdependence of the welfare of human and nonhuman elements in the natural world (Mayer and Frantz, 2004). Authentic Eco-Appreciation corresponds with evidence regarding the positive affective and cognitive implications associated with such connectedness (Howell et al., 2013; Martin et al., 2020; Mayer et al., 2009). Added to connectedness is the suggestion that the depth and effects of the identitial and relational aspects of connecting with nature are hinged upon awareness and fortified by explanatory mechanisms of gratitude and appreciation. Authentic Eco-Appreciation thus focuses on deepening connectedness by fostering relationships that are enriched by conscious recognition of thankfulness.

The second element is based on the acknowledgment that ecological selves (i.e., people’s sense of dependency and identification with other beings sharing life on our planet; Macy, 1990; Matthews, 2006) exist within living ecosystems, and that every organism, development, and event operates within the integrated dimensions of *Place* and *Time*. Place represents spaces in which HNR experiences are shaped. These can be tangible (e.g., homes, neighborhoods, gardens, forests, sea sides, or other regions(, as well as recalled or imagined landscapes, visions, and memories that enable transformation and reframing of experiences. Time can be exemplified through the cycles and rhythms of nature, such as the daily progression of sunrise and sunset, the phases of the moon and the shifting of the nighttime skies, and the changing of seasons. These rhythms serve as powerful symbolic representations that connect us to the basic universal truths inherent to nature. Combining place and time is proposed as essential to noticing HNR and experiencing living, ongoing connections to the broader ecosystem of which we are a part.

The third element is the *Individual-Collective* spectrum, which ranges between individual-nature relations and collective-nature relations and signifies the complexity of human-environment interconnectedness. It highlights the interconnectedness between individuals and both human (e.g., groups, communities, societies) and nonhuman (e.g., plants, animals, places) entities, situating the self within broader socioecological systems. Furthermore, this span represents multilevel viewpoints on person-people-planet experiences and interconnectedness, addressing both collective and ecological selves (Levin and Nevo, 2024; Nevo, 2019).

Guided by these tenets of the Eco-Appreciation Perspective, the question addressed in the present research was: What can be learned from older adults’ accounts of their relations with nature about reciprocal nature-based welfare in older adulthood?

## Method

### Participants and Sampling

Sixty individuals over the age of 65 living in Israel participated in the present study. Table 1 provides detailed information about the sample, in terms of gender, age, and residence in urban or rural locales. All participants were community-dwellers. With regard to geographical distribution, 32% of participants lived in the South of Israel, 38% in the North, and the remainder in its central districts or in Jerusalem. Most participants were married, with 17% widowed. 31% had some form of educational or professional training related to nature and/or the environment.

**Table 1.** Characteristics of the Sample

Residence	Men			Women		
	Age		*N*	Age		*N*
	μ (*SD*)	Min.–Max.		μ (*SD*)	Min.–Max.	
Urban	77.9 (6.59)	65–82	8	74.3 (5.64)	65–84	22
Rural	74.8 (4.66)	70–87	17	72.3 (5.83)	65–85	13
Total	77 (5.41)		25	73.1 (5.71)		35

Six trained research assistants approached older adults at senior day centers across Israel and invited them to participate in the study. Invitations were also widely distributed on relevant social media platforms (local Facebook and WhatsApp groups), soliciting direct participation or referrals of potential participants to the research team. The study was described as addressing older adulthood and the natural environment. Sampling continued until diversity was achieved in gender, age, and geographical distribution.

### Procedure of Data Collection

Interviews were performed by the research assistants, individually and in person, throughout June and October 2022. Perhaps noteworthy is the fact that the interviewers themselves included both younger and older adults. Although age was not originally a criterion for inclusion in the research team, diversity in interviewers’ ages provided a variety of perspectives on the wording of questions posed to interviewees, on sensitivities to be considered, and on interviewers’ own attitudes toward older adults. These were all reflected upon during team meetings and enabled the production of a more nuanced interview protocol. Each interview lasted between 40-80 minutes, was carried out at the location and time of participants’ choice (mostly at home), and was audio-recorded and transcribed for analysis. All the interviews were conducted in Hebrew.

The study received the approval of Tel Aviv University’s ethics committee, and all the participants completed an informed consent form prior to their participation.

### The Research Tool

Interviews were guided by a semistructured protocol, enabling reference to given questions alongside a more conversational atmosphere, encouraging participants to elaborate on the research subject as they saw fit (Kallio et al., 2016). The structured questions, drawn from the Eco-Appreciation Perspective, included: “What is nature to you? How would you define nature? How would you describe your relationship with it?,” “Could you share a significant experience you had in nature? How did it make you feel? Who were you with? When and where did it take place?,” “Looking back at your life so far, have you experienced changes in nature’s presence, roles, or meaning to you?,” “What do you think could contribute to more beneficial relationships between older adults and nature?.” Participants were encouraged to be as detailed and as elaborate as possible in their responses.

### Positionality

The first author is a woman in her 40’s, who lives in a small rural Moshav in Israel’s lowlands. She is highly experienced in conducting nature therapy-based and HNR-based workshops with diverse aged groups—from children to older adults, as well as with professionals working with them. Her research background is rooted in multidisciplined sustainability and focuses on HNR, nature-based welfare, and socioecological systems. The second author is also a woman in her 40’s. She lives in a quiet neighborhood on the outskirts of a large city in the center of Israel. Her perspective on HNR was shaped by extensive traveling as a child and the exposure to diverse appearances of nature and peoples’ relationship with it. Her research deals with conceptual connections among social, epistemic, and eco-social justice, also with specific regard to the status and welfare of older adults. Both authors are long-term carers for older family members.

### Data Analysis and Translation

Analysis was led by the first author. Coding was conducted by her under the supervision of the second author. Advanced stages of thematic refinement were carried out jointly. The analysis was performed using a sequential deductive-inductive approach (Azungah, 2018). It began with using a broad deductive approach, guided by the Eco-Appreciation Perspective and its defining elements. Initial themes were elicited with attention to these elements, and the data was coded into categories based on relevance. For example, uses of relational words to describe nature, displays of thankfulness, or personifications of nature as a friend or companion, were coded as potentially reflecting Authentic Eco-Appreciation; detailed descriptions of physical environments or recollections of childhood experiences with/in nature were coded as potentially reflecting dimensions of Place and Time in participants’ HNR; and “we,” “our” or “us” classifications within participants’ generalized references to older adults and their relations with nature were coded as potentially pertaining to the Individual-Collective spectrum. Overlaps between elements were also noted, for example—described experiences of participants in nature that took place with their families and evoked thoughts about passing on love for nature to future generations were coded as addressing both the notion of time and Individual-Collective relations with nature. Coding was performed with heightened awareness of research trustworthiness, and faithfulness to participants’ narratives was continuously reexamined and ensured.

In the second phase of analysis, key issues detected in the first phase were revisited inductively as per the methodological principles of Thematic Content Analysis (Corbin and Strauss, 2008). The purpose of this was to let data also “speak for itself’ and explore how a more descriptive approach could inform the conceptualization of the Eco-Appreciation Perspective. In this phase, analysis was guided by participants’ narratives surrounding the meanings they themselves had attached to their experiences (Tjora, 2018). This allowed for new insights to develop, and highlighted issues previously not considered to be central to the perspective. For example, the detailed sentimental wording of childhood memories added to our comprehension of the integration of Time and Place with Authentic Eco-Appreciation. It taught us that through the remembrance of specific episodes of engagement with nature, authentic appreciation is not only restored but can also be forged. We observed how the experience of recollection itself can spawn a distinct and new authentic appreciation process, which—when combined with reconstructed memories, multiplies opportunities to enhance older adults” nature-based welfare.

Following both phases of analysis, all categorizations and sub-categorizations were assembled in themes, that were reviewed for conceptual coherence and accuracy in representing participants’ accounts. This was carried out until basic saturation appeared to have been achieved—i.e., no further main themes or categories were detected—and relevant data was fully reflected in the recorded findings (Saunders et al., 2018). Qualitative data analysis software was not used in these processes.

In advanced stages of writing-up the findings, quotes incorporated into this article were translated to English. Each excerpt was translated by the second author and reviewed by a certified language editor, both fluent in written and spoken English and Hebrew, and closely familiar with nuances, euphemisms, and cultural references that can construct speech and meaning in the two languages. Then, edited translations were compared to the original wording in Hebrew by the first author. Consultation between the three was conducted in order to minimize the risk of meaning loss (Yunus et al., 2022), and continued until translations were assessed as expressing the same content and sentiments as the quotes in Hebrew. In two cases (a participant’s rephrasing of a Bible verse, and a term in Yiddish), the full interviews from which quotes were taken had to be revisited for context, and final discrepancies between the translation and the original text were fixed.

The findings appear in the following section; numbers replace participants’ names.

## Findings

### Theme I: *HNR as Presence and Being*

For participants in the current study, nature took on many forms and roles. For some, nature was the backdrop for experiences (e.g., “nature for me is birds, flowers, grass, horizon, trees”; 47), or a space wherein processes of enjoyment are facilitated: “Nature is a place where plants, trees and flowers grow, and which is outside private territory. Everyone can enjoy it, it can be a park, […] or of course outside the city, under the sky” (22). For others, it was an intrinsic part of their being, inseparable from them and from humankind in general. As (52) shared, “We are part of nature. The fact that we change it and influence it does not change the fact that we are part of it.” To the question “What is nature to you, in one word?,” ten participants responded “life.” (45) explained this response, identifying nature as the source of sustenance and growth: “Since we are biological beings, nature for us is the essence of life, it is our air, it is our water, it is our oxygen, it is our food, it is everything that allows us to live.”

Participants described nature as a constant influence in their lives. Although they observed changes in the environment and in humans’ attitudes there toward, its status, and the basic connection to it, were irrefutable. In (24)’s opinion:

In the past we all lived only in nature. And today we have somehow become more disconnected from our environments… and still, there can be bits of nature both outside and inside, inside the mind, inside the consciousness, inside the body is also nature.

The continuity of nature provided participants with a sense of safety and comfort, sometimes offering an alternative to other, harsher contexts. (40) shared that “Nature for me is always a place that brings renewal, innocence, repetition. Whatever happens – in politics, in the world – nature marches to the rhythm of its own drum, according to its own rules,” expressing what can be understood as a sense of existential trust in nature, and in its ability to facilitate peace of mind, perhaps where human society failed to do so.

### Theme II: HNR as Connection

All of the participants maintained some level of conscious relationship with nature. Like (14), many participants emphasized this relationship’s reciprocal character: “What you give to the plant, the plant gives back to you. This is how I feel. The plant gives back to you; it sees that you care about it, that you water it.” (60) felt that in the relationship with nature, there was less scrutiny and more recognition than in other relationships: “[Nature] is the place where I actually feel my soul is most whole, where I’m being appreciated... the criticism is silenced... everything goes through a different lens.” For (3), the relationship with nature is the most fundamental interaction of human existence: “In principle, from the earth you came and to the earth you shall return. So, I have an ongoing dialogue with the earth about when I am returning to it.”

The affiliation with nature, that ran through time and consequences, was depicted as a steady source of “Love, a feeling of connection” (34). Access to its benefits changed over the course of participants’ lives, but the well-being it enabled persisted. (39) told us:

There is some tendency in old age to withdraw… I think the greatest advantage of nature is that it takes us away from screens… it doesn’t have to be a trip to the Niagara Falls... it can be a walk on the path near the house, hearing two or three birds... or even sitting – you don’t have to walk. This thing of being connected to what’s happening outside, in the world of plants, birds, and other animals, for me it’s... ah... it’s really a precondition of feeling good. That’s my well-being.

Activities in nature, cited by participants, were numerous. These included individual and nature-oriented activities (e.g., planting trees, gardening in one’s yard or in a community garden, soil enrichment, ecological restoration, and environmental education), as well as leisure activities, (e.g., outdoor walking, meditation, swimming, breathing fresh air, nature photography/painting, excursions in the outdoors, simply sitting on a balcony or reading near a window with a view of nature, and reading about nature). Activities with direct human-nature relationality, such as cultivating personal openness, sensitivity, awareness, attention, observation, and mindfulness of the natural world were also cited. As, was connecting with others in the context of shared HNR through activities like guided group walks in nature, storytelling, working in community gardens, and jointly promoting nature accessibility. Various living situations enabled diverse opportunities to engage in such activities and sustain relations with nature. Participant (13), shared:

When you’re at home, you feel like everything is closing in on you. And when you go out – walk a bit, wander in nature, even if it’s urban nature, it doesn’t have to be wilderness nature – it simply gives you oxygen, it gives you the strength to go on.

Participant (36), who lives in a rural area, added:

I can’t imagine leaving this view for anything. But people have different preferences. I can’t imagine giving up this direct contact with nature. Even people who move to the big city and walk the streets, they also travel abroad, and then... nature is their leisure time. And for me, nature is there all the time.

Some of the participants’ responses illuminated aspects of their relations with nature that interwove awareness and emotions. For example, (44) mentioned that “The very fact that you love nature, and the fact that you enjoy nature, already contributes to your welfare.” Participant (14) added:

If I plan to go to the nursery today, I have a goal. I don’t just go to the nursery, I go to the nursery because I want to buy flowers and I want to plant them and also seeds, I buy a bunch of seeds and I want to plant them, and I also want them to succeed, for them to bloom, for them to grow, for me to follow their development. And that makes me feel good. When a plant wilts, I wilt along with it. It hurts me. It hurts me that it wilts.

In fact, it seemed that the impact of engagement with nature on participants had to do with the relational meanings that they attributed thereto, no less than with the intensity or physical scope thereof.

### Theme III: HNR as Past, Present, and Future

Relations with nature were deeply rooted in participants’ childhood experiences and upbringing. They mentioned memories of significant adults who contributed to such relations and independent pursuits that enabled special experiences with/in nature. Some of their most joyful memories revolved around family vacations in nature, as shared by (30): “I remember the best time of my life as the holidays, when we would go to my grandparents and we would walk in nature and walk around the orchards, the fields, this... was the happiest time of my life.” Participant (34) added:

Someone who was educated about nature from childhood, from youth at least, I think it’s... take nature away from him, and he’s poor. Tell me today that I can’t walk on some mountain, on some leaf, near some lake... I’m poor. I can’t even think about it.

Participant (31) recalled, “My mother made us very close to nature... it’s things she would say, like ‘Look, look at this common blackbird [Turdus merula].’ She really... through her I learned to look at birds and look at the trees.” Some mentioned that when adult presence was missing in their lives, nature partially filled their need for affection and attention:

My childhood was not so easy… our household fell apart a bit, the parents… and I was blessed with the field… there I would escape to, both on the way to school and on the way from school… I would look at bumblebees then, sitting on the ground and watching them and following them, and I’d forget myself. And there was one flower that I always waited at, for one bumblebee. (50)

Participants reported that as they aged, their relations with nature gave them perspective and a sense of harmony surrounding thoughts about aging, passing, and death: “Like seeing the flowers wither or the trees dry up or the rocks fall from the cliff, the seasons change... so also in the body... change, aging, growth. Such a great cycle, here it happens and there it happens” (24).

However, older adulthood also gave participants an outlook on changes in humankind’s relationships with nature. (45) described feeling depressed when contemplating future generations facing urbanization and extinction of nature:

I don’t want to think about it. […] we humans are multiplying at an enormous rate, the living space on earth is limited, and humans begin to close themselves in “small boxes”... we build skyscrapers... and man moves away from the earth, and a person moves away from nature, and a person moves away from himself, because as soon as you start moving away from nature and withdrawing, you move away from humanity. You move away from humanity, you move away from a lot of things... So I don’t want to think about what will be the natural future of future generations.... that’s why I told you that I feel anxious about the world my granddaughters were born into.

Participant (46) expressed pessimism and worry:

The climate crisis, and the talk about it, started a few years ago, and the talk is increasing, and also the actions that are being taken too little and too late. Maybe something will change, maybe. But the future as I see it as very bad. And to some extent I welcome the fact that I won’t be here when this thing comes. My heart – my heart goes out to my children and my grandchildren, but oh... it will be a very bad world.

Placing HNR and the experiences that nurture it, and are in turn nurtured by it, on a temporal continuum, evoked both comfort and concern; a duality seemingly interlaced with transformations in the natural environment, in humankind’s relations with nature, and in participants’ own bodies and minds as aging took place.

### Theme IV: HNR as Benevolence

“People need to go outside. Mainly—as a legacy for their children. But when they go out, the most important thing—as I’ve already said—they must know it [nature], love it, and respect it.” (37)’s words, that echo a sentiment shared by many participants, emphasize the acknowledgement of nature as a subject, an entity worthy of gratitude. Such gratitude was described as a source of welfare in itself, and as an important driver of the survival of humankind. Some participants viewed appreciating nature as moral act, of doing right by nature after all that nature’s done for them (43):

Nature welcomed us into it and not the other way around. And you can’t spit in nature’s face... It accepted you, it made room for you, you arrived. You’re a thinking creature, you started creating all kinds of things and all kinds of inventions... and slowly you started harming it. That is not appreciating.

Notably, the ability to continue to find moments and avenues for valuing nature was cherished by participants. It provided a much-needed platform for feeling worthwhile, and for finding meaning in who they are and what they do: “I feel that nature gives to me, and I give to it. I greatly respect nature. I sometimes go to it with a feeling of great privilege” (50). This acknowledgement, shared by several participants, was central to their ability to maintain HNR and experience its benefits as the years went by, and was consistently communicated as not taken for granted in older adulthood.

## Discussion

The findings of the present study offer some novel insights into older adults’ reciprocal nature-based welfare, as it is reflected in descriptions of their current and changing relationships with nature. First, it can be seen that for participants in the current study, HNR and the options that it provides for enhancing their welfare were ardently sought. Although a great deal of the literature on HNR and older adults addresses interventions and therapeutic processes that entail bringing older adults to nature or bringing nature to older adults, the findings of the present study uncover the many opportunities that older adults identify and realize to engage with nature, even in their immediate environment, and even indoors. This implies that previously gained insights regarding the many benefits of positive HNR on cognitive, social, affective, physical, and behavioral aspects of older adulthood should not only be applied to enabling structured interactions of older adults with/in nature, but can also be used to build on the many alternatives for experiencing such relations as they present themselves in older adults’ daily lives. Clearly, the more participants’ engagement was characterized by awareness of the sensory facets of nature (e.g., visual beauty, singing birds), and/or involved caring for nature (e.g., growing a plant), the more powerful—mostly positive—emotions it evoked. These emotions were yet enhanced in light of gratitude toward the very act of engaging with nature, whether actively or passively. This suggests that appreciation is not only a pleasant byproduct of HNR among older adults, but is a rather a factor to explicitly consider and develop when searching for available venues for enhancing and maintaining their welfare.

Second, contemplating how HNR transpired over the course of their lives enabled participants to reexperience positive feelings attached to with/in nature, even when recalling memories of childhood absence or loss. Working with recent memories has been found useful in deepening the understanding of individuals’ and communities’ relations with nature (O’Donoghue, 2006). The present study confirms that delving into early memories can not only do the same but also provides some of the advantages of experiencing positive HNR in older adulthood. Our findings echo, in part, what is known about reminiscence therapy, that links prompting (mostly) verbal recollections of life events with improved moods, self-forgiving, finding meaning, an increase in seeking social connections, and reconciling past and present identities (Bhar, 2014; Cuevas et al., 2020; Yang et al., 2018). They add to some recent research on nature-based reminiscence interventions (e.g., Lee et al., 2020), suggesting that specifically reviewing authentic episodes of appreciation or raising awareness of appreciation aspects of past experiences with/in nature may be especially helpful toward accessing internalized sources of resilience and increasing nature-based welfare in older adulthood. When addressing their present age and situation, acknowledging the reciprocal elements of NHR encouraged participants to reaffirm a sense of agency and purpose. Nature was often subjectified in interviews, anthropomorphized as a partner that enabled participants to fulfill roles of which human society often deprived them. In their interpretation, nature readily welcomed their presence, even if this presence was more limited, or looked different, than when they were younger. Furthermore, their acquaintance with, knowledge of, and respect toward nature, proved advantageous in their relations with it, contrary to ageist attitudes often faced by older adults, that position their knowledge and experiences of NHR as irrelevant and outdated (Levin & Nevo, 2024).

Although thinking about the future, HNR was a dominant element in what participants could name as their legacy, adding another layer of meaning thereto. Some wanted to impart their appreciation for and reverence of nature to their offspring, perhaps also as a way to emphasize the need to care for nature after their passing. The cyclical traits of nature assisted some participants in framing their own mortality in terms of acceptance, reflecting the fact that we are all subordinate to nature’s progression. This was not a focal point of our research and hence was not developed further. Additional research is accordingly encouraged on the effects of HNR, appreciation, and recollection, on attitudes toward dying and the end of life.

Third, to many participants, HNR facilitated feelings of belonging and collectiveness. In HNR, their age was not a source of deprivation or of discrimination. Rather, it was just part of being; their position within the ecosystem was not something they had to justify nor prove; it was a given and consistent aspect of their existence. It was always there, and when discussed and made aware of, conjured genuine emotions of love, affection, and affiliation. Previous research has shown the immense importance of sense of community to older adults’ ability to weather some of the challenges of aging (Buckley, 2022). Broadening the scope of reference regarding community bonds and identities in older adulthood to include nonhuman relationships, opens up opportunities to redefine, enhance, and promote older adults’ welfare.

The application of an Eco-Appreciation Perspective and its tenets (Nevo, 2019) enables the exposure of typically overlooked nuances concerning the development of welfare in older adulthood, which stem from an eco-centric point of view on contemporary aging. These nuances, and the principles of Eco-Appreciation, can be helpful to professionals and policymakers charged with promoting older adults’ safety and quality of life. Focusing on appreciation’s role in shaping older adults’ welfare also introduces new ways of thinking about and applying other, possibly neighboring, concepts such as transcendence and self-transcendence (Worth & Smith, 2021). Transcendence in later life, or gerotranscendence, is referred to as a developmental shift in one’s mind-set “from a materialistic and rational view of the world to a more mystical or cosmic perspective leading to redefinition of time, space, life, death, and the self” (McCarthy & Bockweg, 2013, p. 86), often accompanied by growing awareness, the expansion of mental boundaries, and a sense of awe (Abreu et al., 2022; Bethelmy & Corraliza, 2019). Reviewing this notion considering the present study’s findings underscores the dynamics between contemplation about the vastness of nature and a specific form of mental reframing that is appreciation- and relation-based; and can be fostered by particular and accessible daily episodes of attention toward HNR.

The present research not only emphasizes the active role that older adults can play in deepening their connection with nature, but also calls on professionals in health, gerontology, and community care to recognize and harness the mutual benefits of appreciating this bond. As we face looming environmental challenges, the unique wisdom, perspective, and experiences of older generations, along with professional acknowledgment of nature’s value to older adults’ well-being, offer invaluable insights. Accordingly, embracing an ecological appreciation perspective opens new pathways to enhancing both individual and collective resilience.

Some of the study’s limitations should be taken into account. First and foremost, despite persistent attempts, we failed to assemble a sample that was ethno-nationally diverse. The fact that data were collected during violent clashes between Israel and Gaza and among Jewish and Palestinian citizens of Israel plausibly negatively affected the availability to participate in our study. This was sporadically relayed by Arab-Palestinians who chose to forgo participation. Research into the experiences and valuable insights of older adults from Arab communities in Israel is essential to gain a more complete response to the research question and to refer to the intersection among culture, HNR, appreciation—and perhaps conflict. Also, it is possible that participant self-selection took place, and that those for whom HNR were more positive were more inclined to participate in the study. Although this does not detract from the validity of their narratives, it may explain why although asked about, negative experiences with/in nature were scarcely mentioned. Future research can focus on such aspects and continue exploring experiences of eco-appreciation in the many diverse contexts of older adults’ lives.

## Data Availability

Raw data from this study is not available due to IRB and ethical restrictions.

## Author Note

1. The term “welfare” applied throughout this article is defined as “the measure of how well someone’s life is going for them” (Fletcher, 2022); operationalized as encompassing the social, physical, emotional, and financial status of older adults—as individuals and as a distinct social group, and the ability of nature to flourish (Vucetich et al., 2015). It aligns with the term “nature-based welfare,” which we suggest draws attention not only to well-being derived from HNR but also refers to the broader advantages of expanded consciousness and deeper existential reflection.
